# Characterizing the chloroplast genome of *Mammillaria elongata* DC. 1828 in the Cactaceae family and unveiling its phylogenetic affinities within the genus *Mammillaria*

**DOI:** 10.1080/23802359.2023.2265100

**Published:** 2023-10-11

**Authors:** Yang Ni, Jingling Li, Qianqi Lu, Haimei Chen

**Affiliations:** a Center for Bioinformatics, Institute of Medicinal Plant Development, Chinese Academy of Medical Sciences, Peking Union Medical College, Beijing, PR China; b College of Horticulture and Landscape Architecture, Southwest University, Chongqing, PR China

**Keywords:** Chloroplast genome, gene loss, genome size variation, *Mammillaria elongata*, phylogenetic analysis

## Abstract

With its nearly 200 species, the *Mammillaria* genus is the most species-rich within the Cactaceae family, yet surprisingly, few of its chloroplast genomes have been studied. We focused on the species *Mammillaria elongata* DC. 1828, a petite cactus native to Mexico and favored by horticulturists, yet whose phylogenetic relationships remain uncertain due to a lack of genomic data. We extracted the DNA from a sample obtained in China, sequenced it using the NovaSeq 6000 platform, and assembled the chloroplast genome using GetOrganelle software. Our assembly resulted in a chloroplast genome of 110,981 base pairs with an overall GC content of 36.28%, which included 100 genes (95 unique). Notably, several protein-coding genes were absent. Phylogenetic analysis using 59 shared genes across nine *Mammillaria* species and one Obregonia species revealed that *M. elongata* and *M. gracilis* are closely related, suggesting a recent common ancestor and possible shared evolutionary pressures or ecological niches. This study provides crucial genomic data for *M. elongata* and hints at intriguing phylogenetic relationships within the *Mammillaria* genus.

## Introduction

Situated within the Caryophyllales order, the cactus family (Cactaceae) graces the horticultural world with its captivating ornamental plants. This extensive family encompasses about 174 genera and nearly 2000 species (Novoa et al. 2014; Abouseadaa et al. 2020). Historically, the taxonomic classification of Mammillaria (Cactaceae) has been challenged by extensive morphological variation and species sympatry. Through the deployment of chloroplast markers, Cristian R. Cervantes et al. have advanced our understanding by constructing a detailed phylogenetic tree, thereby refining the infrageneric classifications, encompassing subgeneric, sectional, and series categorizations (Cervantes et al. 2021). Among this remarkable biodiversity, the *Mammillaria* genus stands out with almost 200 universally accepted taxa, marking it as the genus with the greatest number of species (https://cactiguide.com/cactus/?genus=Mammillaria). One of the most cherished species within this genus is *M. elongata*. This petite cactus is easily propagated and favored among plant enthusiasts. Native to Mexico, it prospers in Guanajuato, Hidalgo, and Querétaro states. It is adapted to altitudes that range from 1300 to 2300 m above sea level, demonstrating its ability to flourish under such conditions (Breslin et al. 2021). Despite the extensive use of chloroplast genomes for phylogenetic analysis, there is a surprising scarcity of publicly available data for *Mammillaria* species on the NCBI database. Despite the genus’s rich diversity, only a handful of *Mammillaria* chloroplast genomes have been unveiled. Consequently, the phylogenetic relationships of *M. elongata* remain shrouded in uncertainty.

## Materials and methods

**Figure 1.**
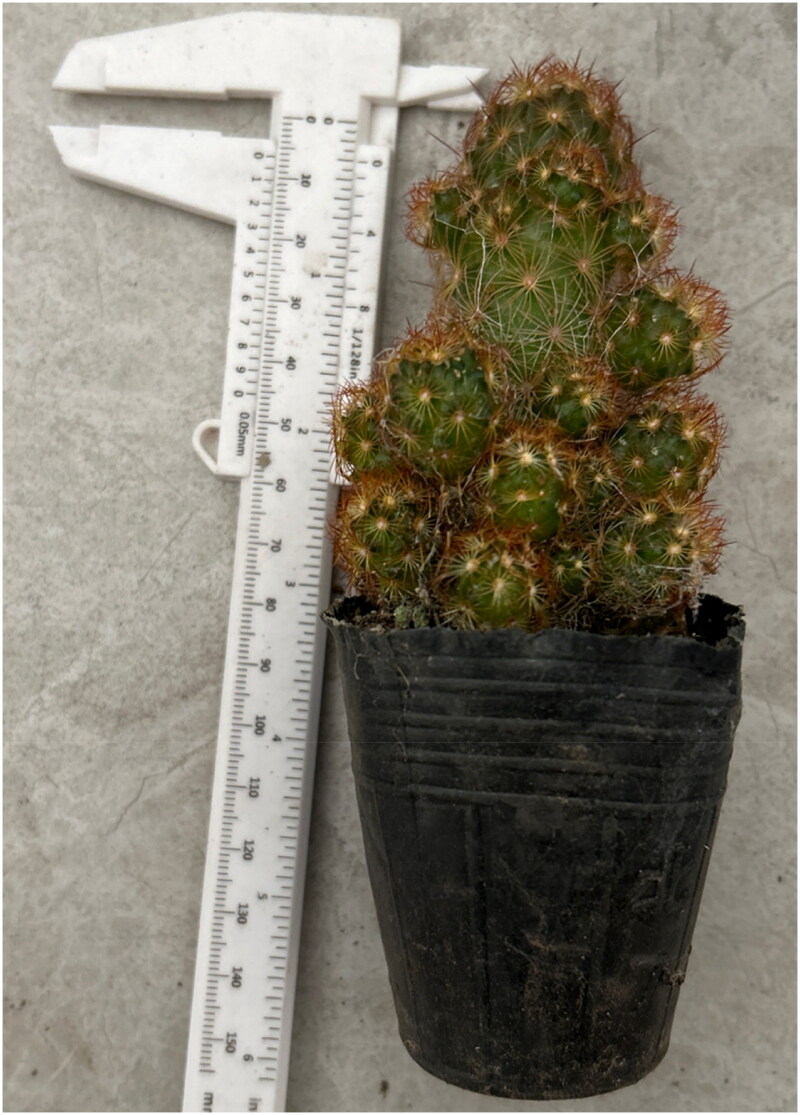
Photographs of *M. elongata* (the photograph were taken by prof. Haimei Chen**).** the image was captured at the Horticulture and Landscape Architecture College of Southwest University, Chongqing, China. The geospatial reference for this location is N29.842889, E106.394527. This visual representation demonstrates the distinctive features of the compact cactus, which is native to Mexico and highly appreciated for its ease of propagation in horticultural practice.

We selected the sample from China to explore genetic and evolutionary differences in the chloroplast genome between this sample and the native *Mammillaria* populations in Mexico, further studying the adaptive evolution of *M. elongata*. Our sampling site was at the College of Horticulture and Landscape Architecture, Southwest University, No. 2 Tiansheng Road, Chongqing, China, 400716. The site’s geospatial coordinates are N29.842889, E106.394527 (Figure 1). We employed a Magnetic Plant Genomic DNA Kit (DP342, Tiangen, China) for DNA extraction. The subsequent library construction was carried out using the NEB Next Ultra DNA Library Prep Kit for Illumina (NEB, Ipswich, MA), and an insert size of 350 bp was achieved. Sequencing procedures were conducted on the NovaSeq 6000 platform. The plant samples stored at the herbarium of the Institute of Medicinal Plant Development with the accession number Implad ME01 (Institute website: http://www.implad.ac.cn/, Contact person: Yang Ni, E-mail: ny_work@126.com).

The chloroplast genome was directly assembled *de novo* from this raw data, utilizing the GetOrganelle software with its default settings (Jin et al. 2020). The assembled genome was assessed rigorously: original sequencing results were aligned with the reference genome using BWA software (Li and Durbin 2009), genome coverage depth was computed using Samtools (Li et al. 2009), and a line graph was constructed using a customized Python script (https://www.protocols.io/view/generating-sequencing-depth-and-coverage-map-for-o-4r3l27jkxg1y/v1). The Gepard software aided in verifying the assembly’s start point and direction accuracy (Krumsiek et al. 2007). Upon verification, we performed genome annotation using the CPGAVAS2 software and data set 2 (Shi et al. 2019), with the annotation results subsequently inspected and visualized using CPGView (Liu et al. 2023). Any detected annotation inaccuracies were manually corrected with the Apollo (Lewis et al. 2002) software. The final annotated results were submitted to the NCBI database through the Bankit software.

Phylogenetic analysis was initiated by downloading eight chloroplast genomes from the NCBI database. Shared genes were extracted using PhyloSuite software (Zhang et al. 2020), and the identified gene clusters underwent multiple sequence alignments using MAFFT (Katoh and Standley 2013). A Maximum Likelihood (ML) tree was subsequently built using the IQTREE2 software (Nguyen et al. 2015), adopting the best-fit model as per the Bayesian Information Criterion (BIC), which was GTR + F + I + G4. Branch supports were evaluated using the ultrafast bootstrap method with 1000 replicates (UFBoot) (Hoang et al. 2018).

## Results

**Figure 2.**
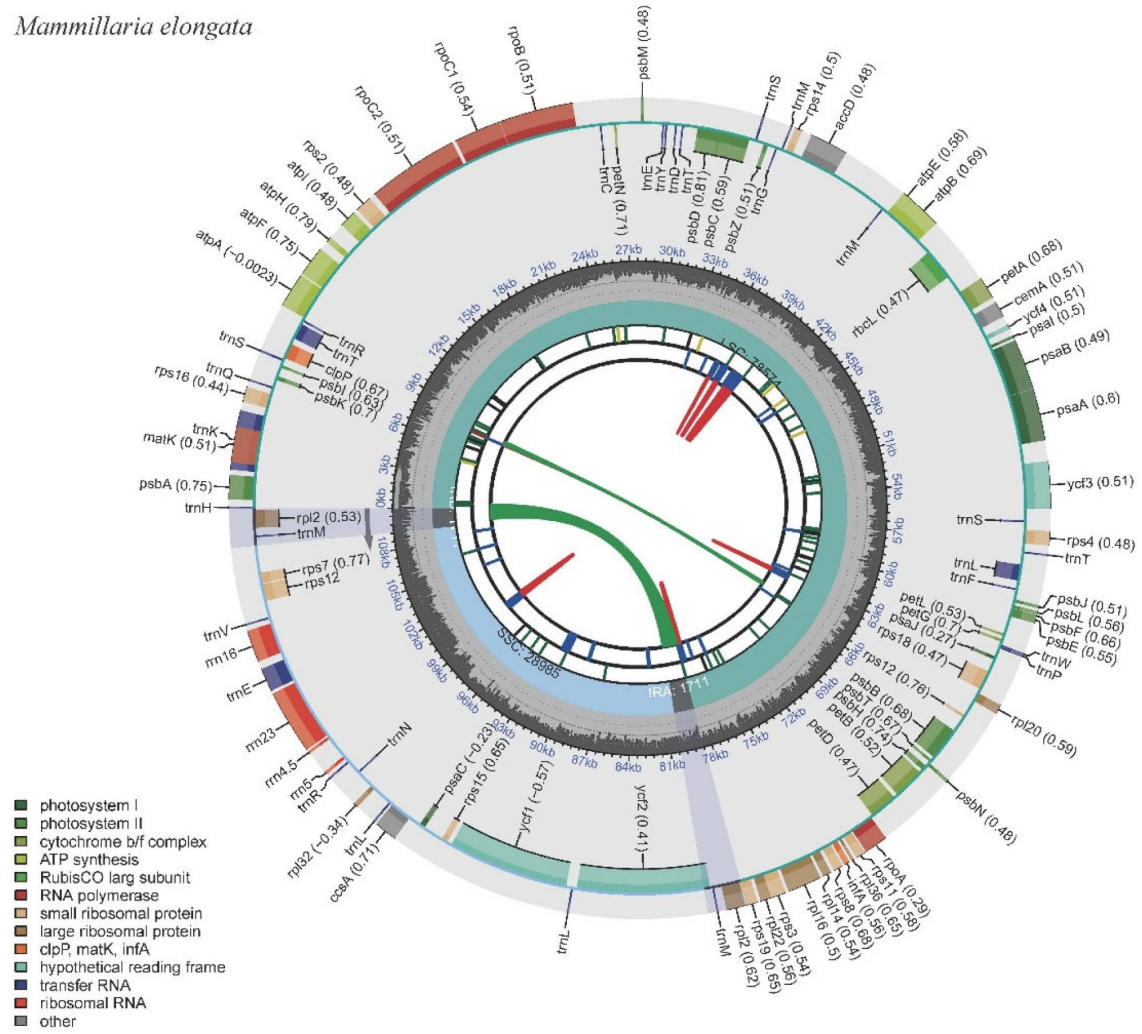
The chloroplast genome of *Mammillaria elongata* visualized by CPGView. The outer circle depicts the locations of protein-coding genes, tRNA genes, and rRNA genes along the 110,981 bp circular chloroplast chromosome. Genes are color-coded by function: photosystem I (orange), photosystem II (red), cytochrome b/f complex (purple), ATP synthase (blue), rubisco large subunit (green), RNA polymerase (teal), ribosomal proteins (small subunit in pink, large subunit in grey), other (light blue). The inner circle indicates the genome position in kilobase pairs.

The *M. elongata* chloroplast genome spans 110,981 base pairs (bp), featuring an overall GC content of 36.28%. The genome assembly quality was assessed. The average sequencing coverage depth is 2396.22x, with a maximum depth of 3586x and a minimum of 921x (Figure S1). The genome is structurally organized into four distinct regions: two inverted repeats (IR) regions, each 1711 bp long with a GC content of 38.05%; a small single copy (SSC) region spanning 28,985 bp and boasting a GC content of 38.06%; and a large single copy (LSC) region, which is 78,574 bp in length and has a GC content of 35.55% (Figure 2; Table S1).

This chloroplast genome encompasses 100 genes, of which 95 are unique. The genes comprise 67 protein-coding genes (with 66 of them being unique), 29 tRNA genes (of which 25 are unique), and four rRNA genes, all unique (Table S2). Notably, several anticipated protein-coding genes are absent. The absent genes include the complete suite of subunit genes for the NADH-dehydrogenase complex (namely *ndhA, ndhB, ndhC, ndhD, ndhE, ndhF, ndhG, ndhH, ndhI, ndhJ,* and *ndhK*), as well as two large ribosomal subunit proteins, *rpl23* and *rpl33*. The genome also lacks the conserved open reading frame, *ycf15*. Embedded within the genome are 13 genes that carry introns (Figure S2). Among these, 11 genes harbor a single intron and two genes with two introns (Figure S3; Table S2). Interestingly, the *rpl2* gene has lost its intron. To summarize, most intron-carrying genes in this chloroplast genome possess one intron, two genes contain two introns, and the intron typically present in the two copies of the *rpl2* gene is absent (Table S2).

**Figure 3.**
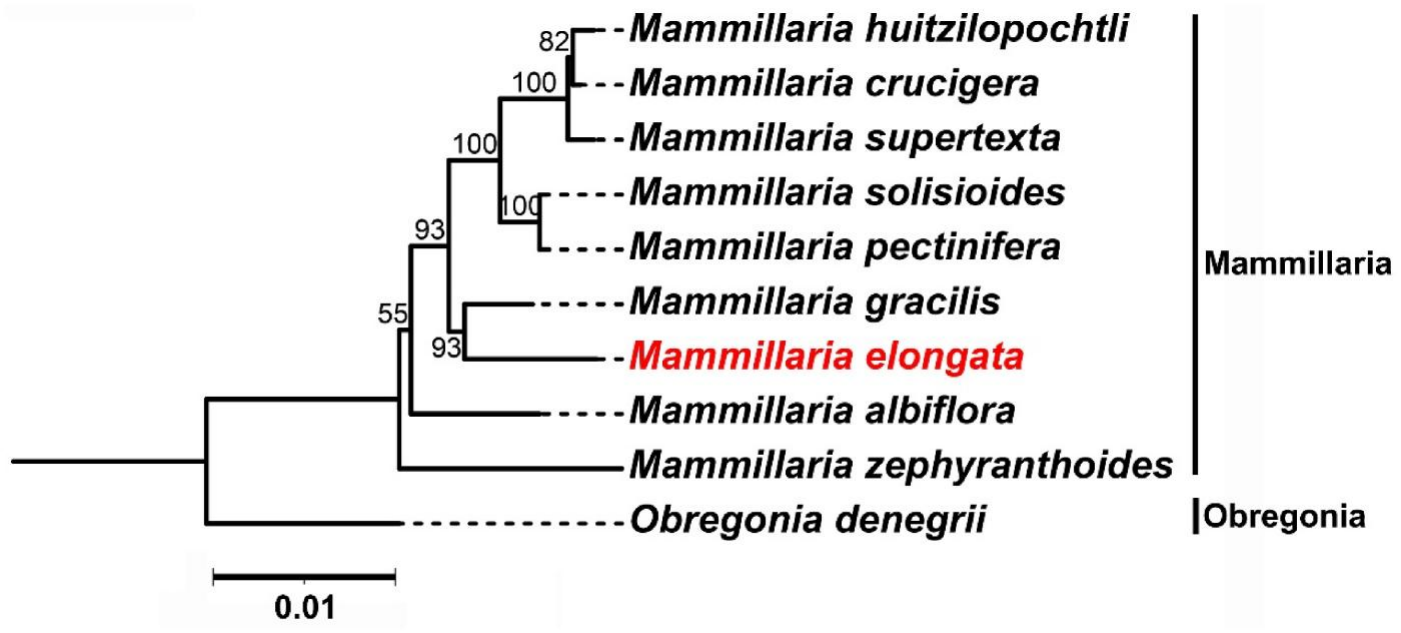
Maximum Likelihood (ML) phylogenetic tree Illustrating the relationship between *M. elongata* and eight other species within the *Mammillaria* genus based on chloroplast genome sequences. *Obregonia* species are utilized as outgroup references. *M. elongata* (MW553058.1) is distinctively marked in red. The sequences incorporated in the tree are as follows: *M. huitzilopochtli* (MN517612.1) (Solórzano et al., 2019), *M. supertexta* (MN508963.1) (Solórzano et al., 2019), *M. solisioides* (MN518341.1) (Solórzano et al., 2019), *M. pectinifera* (MN519716.1) (Solórzano et al., 2019), *M. gracilis* (MW553059.1) (Yu et al., 2023), *M. albiflora* (MN517610.1) (Solórzano et al., 2019), *M. zephyranthoides* (MN517611.1) (Solórzano et al., 2019), and *O. denegrii* (MW553062.1) (Yu et al., 2023). the scale bar represents a genetic distance of 0.1 substitutions per site.

We conducted a phylogenetic analysis using 59 shared genes across nine *Mammillaria* species and one *Obregonia* species (serving as an outgroup) (Yu et al., 2023). In the resulting phylogenetic topology, *M. elongata* and *M. gracilis* are distinctly clustered. This grouping suggests a close genetic relationship between these two species, which may point to a relatively recent common ancestor. Furthermore, this close genetic relationship could suggest that these species have experienced similar evolutionary pressures or inhabited shared ecological niches. Future research focusing on these genetic connections and their ecological implications could further elucidate the biodiversity and conservation needs of this significant group of cacti (Figure 3).

## Discussion and conclusion

Within the Caryophyllales order, the typical length of chloroplast genomes hovers around 150–160 kb, with the IR region generally spanning approximately 25,000 bp (Downie et al. 1997; Choi et al. 2018; Yang et al. 2019). However, within the cactus genus *Mammillaria*, the chloroplast genome of *M. elongata* stands at a mere 110,981 bp. The chloroplast genome size is notably shorter than the chloroplast genomes found in other species of the Caryophyllales order, suggesting multiple genomic contractions and expansions throughout its evolutionary history (Chincoya et al. 2020). The canonical structure of chloroplast genomes is quadripartite. Intriguingly, the IR region in the *M. elongata* chloroplast genome is just 1,711 bp, significantly less than the standard 25,000 bp. This phenomenon points to the contraction and expansion events within the IR region as a likely major contributor to the size variation of the *M. elongata* chloroplast genome. This observation aligns with previous reports on *M. gracilis* from the same genus, which also exhibited a significantly contracted IR region, measuring only 1693 bp (Solórzano et al., 2019; Yu et al., 2023).

The *ndh* genes are pivotal in encoding the NADH complex within the chloroplast, instrumental to photosynthesis and vital for plant growth and maturation (Krause 2008). Intriguingly, our genomic annotations spotlight a notable void of the *ndh* gene cluster in *M. elongata*. While prior studies underscore that this deficiency is not comprehensive, traces of some *ndh* gene fragments remain discernible (Yu et al., 2023). Furthermore, the scientific realm is embroiled in a discourse debating if the *ndh* genes faced an all-encompassing loss or gradual attrition throughout evolutionary periods (Ranade et al. 2016), emphasizing the need for more exhaustive research. Evidence from both the Orchidaceae family and the parasitic plants of the genus *Cuscuta* in the Convolvulaceae family points to the varying degrees of *ndh* gene loss (Kim et al. 2015; Ni et al. 2021). One hypothesis posits that the apparent loss of the *ndh* gene family may stem from the transfer of their expression to the nuclear genome. This transfer would then allow these genes to remain functional, albeit from a different genomic location (Stegemann et al. 2003). Supporting this hypothesis are observations from the nuclear genome of *Phalaenopsis aphrodite*, where complete sequences of *ndhA*, *ndhF*, and *ndhH* have been identified (Cai et al. 2015). This suggests the potential relocation of the original *ndh* genes to the nuclear genome. To summarize, the observed loss or diminution of *ndh* genes in certain plant species appears to be a multifaceted process, potentially entailing gene transfer events as well as ecological and evolutionary adaptations.

The chloroplast genome has consistently emerged as a pivotal molecular marker in conservation-focused studies pertaining to cacti. For example, investigations centered on the *Copiapoa* genus, which is native to the Chilean Atacama Desert, harnessed data from chloroplast sequences coupled with microsatellite analyses (Fava et al. 2020). This dual-data approach facilitated a comprehensive assessment of the distribution and potential extinction vulnerabilities of particular taxa, unearthing pronounced extinction susceptibilities for certain members within the genus (Larridon et al. 2018; Fava et al. 2020). A parallel study, honing in on the *Sclerocactus* species, underscored the importance of integrating nuclear microsatellites with chloroplast DNA sequence data. This integrated method proved instrumental in discerning genetic variances and in pinpointing minimal hybridization occurrences between distinct species (Schwabe et al. 2015). Collectively, these endeavors reinforce the instrumental role of the chloroplast genome of cacti in advancing conservation biology. Building on this foundation, our exploration of *M. elongata* paves the way for broader applications of chloroplast genomics, particularly in elucidating and safeguarding the genetic fabric of cacti species under threat of extinction. However, it’s important to note that our samples come from China, whereas *M. elongata* is originally from Mexico, which might affect the reflection of its native genetic diversity.

We sequenced and analyzed the complete chloroplast genome of the *M. elongata* cactus for the first time. The genome is about 111,000 bp long and contains 100 genes, 95 of which are unique. Some genes were found missing in this chloroplast genome. Additionally, our research showed that *M. elongata* and another cactus species, *M. gracilis*, are closely related, suggesting they may share a recent common ancestor and similar evolutionary histories or habitats. This work provides valuable genetic data for further study of *M. elongata* and can help us understand cacti’s evolution and conservation needs in the *Mammillaria* genus and beyond.

## Supplementary Material

Supplemental Material

## Data Availability

The genome sequence data supporting this study’s findings have been deposited in the NCBI GenBank and can be accessed at https://www.ncbi.nlm.nih.gov/ with the accession number MW553058.1. Corresponding BioProject, Sequence Read Archive (SRA), and BioSample entries are available under the identifiers PRJNA995908, SRR25317666, and SAMN36509928, respectively.
