# Supplementary material for: Characterizing the chloroplast genome of Mammillaria elongata DC. 1828 in the Cactaceae family and unveiling its phylogenetic affinities within the genus Mammillaria
Source: Mitochondrial DNA B Resour. 2023 Oct 11;8(10):1071–6. doi: 10.1080/23802359.2023.2265100 (PMC10569349; doi:10.1080/23802359.2023.2265100)
Supplement: Supplemental Material [file TMDN_A_2265100_SM9842.docx]

**Supplementary Figures and Tables**

**
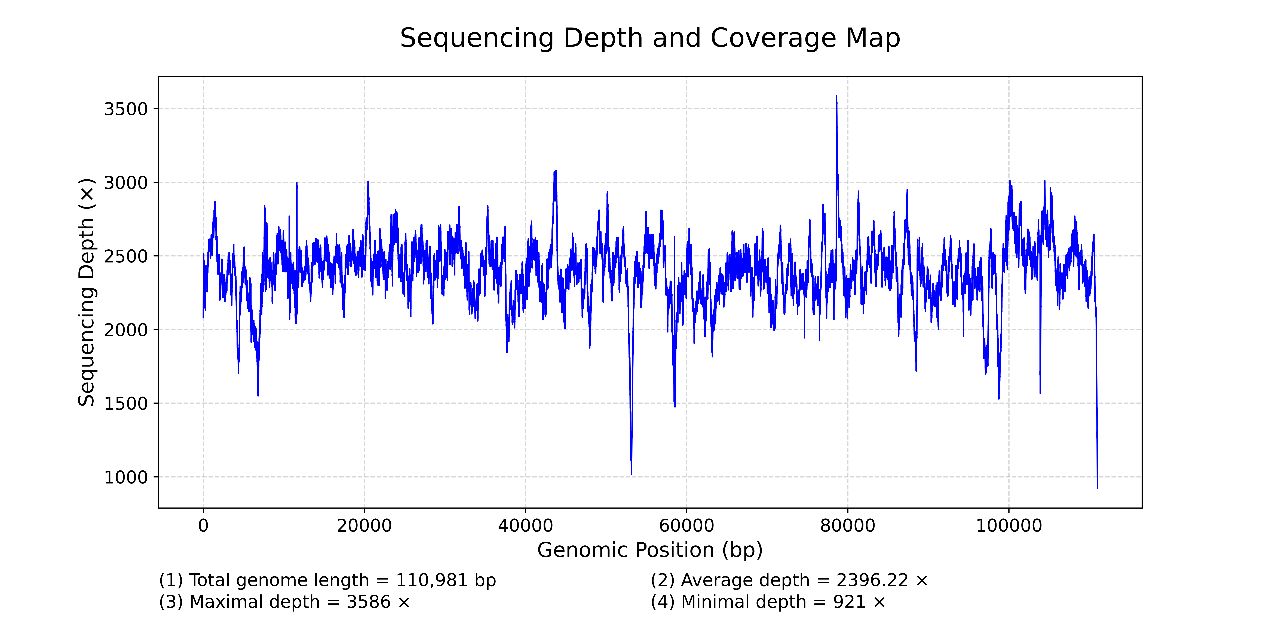
**

**Figure S1.** Sequencing depth and coverage across the genome. The figure shows the sequencing depth (number of reads covering each genomic position) across the full length of a 110,981 bp genome. The average sequencing depth is 2396.22x with a maximum depth of 3586x and a minimum of 921x. The high average sequencing depth indicates good overall coverage across the genome. The maximal and minimal depth indicate some regional variability, but overall the depth is fairly consistent with no large gaps in coverage. This suggests the sequencing provided good genome-wide coverage for variant calling and other genomic analyses.


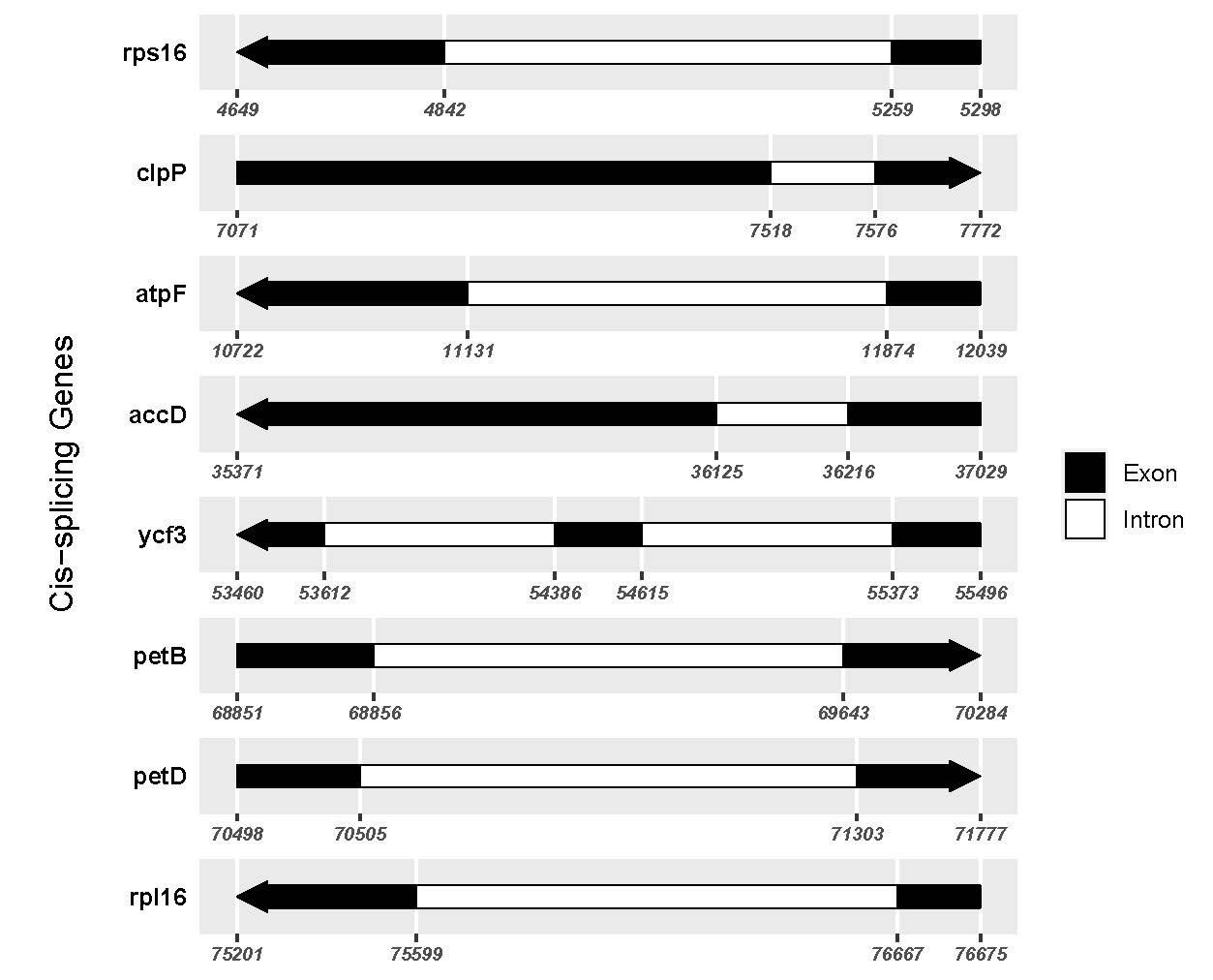


**Figure S2. Cis-splicing genes identified in the *M. elongata* chloroplast genomes.** The table lists cis-splicing genes found in the chloroplast genomes of various plant species. For each gene, the exon and intron nucleotide positions are shown. Six protein-coding genes (*rps16, clpP, atpF, accD, ycf3, petB, petD, rpl16*) that contain cis-splicing introns are included.


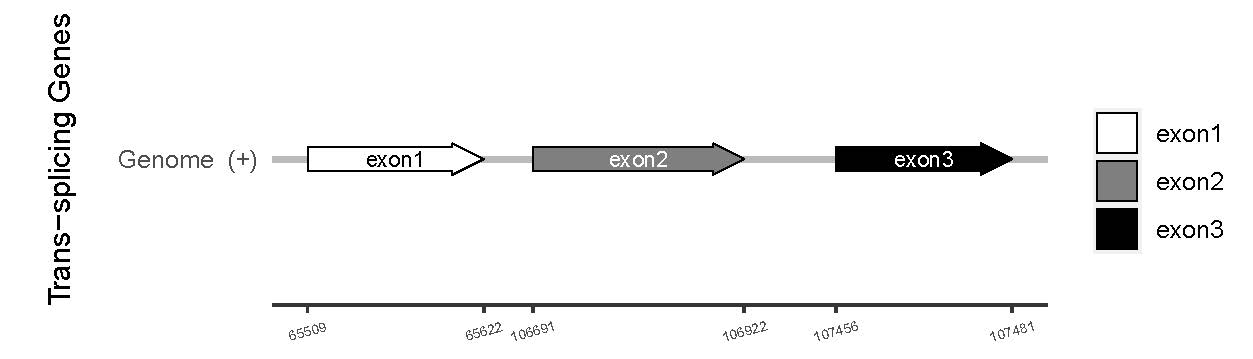


**Figure S3. Trans-splicing genes identified in the *M. elongata* chloroplast genomes.** The diagram shows the exon positions for two trans-splicing genes on the plus strand of the chloroplast genome. For Gene 1, exon 1 is located at position 65509, exon 2 is located at 106691-107456, and exon 3 is located at 65622. For Gene 2, exon 1 is located at 106922, exon 2 is at 107481, and exon 3 is at the same position as Gene 1. This demonstrates how exons from separate genes can be spliced together to form a mature mRNA transcript.

**Supplementary Tables**

**Table S1 Statistics on the Structural Information of the *M. elongata* Chloroplast Genome.**

|  | **Start** | **End** | **Length** | **GC Content (%)** |
| --- | --- | --- | --- | --- |
| Total Genome | 1 | 110981 | 110,981 | 36.28 |
| Inverted Repeat A | 78575 | 80285 | 1711 | 38.05 |
| Inverted Repeat B | 109271 | 110981 | 1711 | 38.05 |
| Short Single Copy | 80286 | 109270 | 28985 | 38.06 |
| Long Single Copy | 1 | 78574 | 78574 | 35.55 |

**Table S2 Exon Locations of the Genes in the *M. elongata* Chloroplast Genome**

| Genes | Exon1 Location | Exon2 Location | Exon3 Location |
| --- | --- | --- | --- |
| accD | [36216:37029](-) | [35371:36125](-) | NA |
| atpA | [9122:10645](-) | NA | NA |
| atpB | [40461:41957](-) | NA | NA |
| atpE | [40057:40464](-) | NA | NA |
| atpF | [11874:12039](-) | [10722:11131](-) | NA |
| atpH | [12460:12705](-) | NA | NA |
| atpI | [13430:14173](-) | NA | NA |
| ccsA | [94529:95509](-) | NA | NA |
| cemA | [46229:46918](-) | NA | NA |
| clpP | [7071:7518](+) | [7576:7772](+) | NA |
| infA | [73782:74027](-) | NA | NA |
| matK | [1989:3527](-) | NA | NA |
| petA | [45009:45971](-) | NA | NA |
| petB | [68851:68856](+) | [69643:70284](+) | NA |
| petD | [70498:70505](+) | [71303:71777](+) | NA |
| petG | [61619:61732](+) | NA | NA |
| petL | [61351:61446](+) | NA | NA |
| petN | [26577:26666](+) | NA | NA |
| psaA | [50289:52541](-) | NA | NA |
| psaB | [48059:50263](-) | NA | NA |
| psaC | [93718:93963](+) | NA | NA |
| psaI | [47824:47934](-) | NA | NA |
| psaJ | [62470:62610](+) | NA | NA |
| psbA | [381:1442](-) | NA | NA |
| psbB | [66377:67903](+) | NA | NA |
| psbC | [31430:32851](+) | NA | NA |
| psbD | [30421:31482](+) | NA | NA |
| psbE | [60243:60494](-) | NA | NA |
| psbF | [60115:60234](-) | NA | NA |
| psbH | [68454:68693](+) | NA | NA |
| psbI | [6605:6715](+) | NA | NA |
| psbJ | [59705:59827](-) | NA | NA |
| psbK | [5999:6178](+) | NA | NA |
| psbL | [59971:60087](-) | NA | NA |
| psbM | [27802:27903](-) | NA | NA |
| psbN | [68248:68379](-) | NA | NA |
| psbT | [68076:68183](+) | NA | NA |
| psbZ | [33556:33744](+) | NA | NA |
| rbcL | [42676:44118](+) | NA | NA |
| rpl14 | [74697:75062](-) | NA | NA |
| rpl16 | [76667:76675](-) | [75201:75599](-) | NA |
| rpl2 | [78645:79469](-) | NA | NA |
| rpl20 | [64326:64724](-) | NA | NA |
| rpl22 | [77501:77947](-) | NA | NA |
| rpl2 copy2 | [110087:110911](+) | NA | NA |
| rpl32 | [96613:96807](-) | NA | NA |
| rpl36 | [73556:73669](-) | NA | NA |
| rpoA | [71908:72936](-) | NA | NA |
| rpoB | [21621:24833](-) | NA | NA |
| rpoC1 | [19549:21591](-) | NA | NA |
| rpoC2 | [15367:19380](-) | NA | NA |
| rps11 | [73008:73424](-) | NA | NA |
| rps12 | [65509:65622](+) | [106691:106922](+) | [107456:107481](+) |
| rps14 | [34609:34911](-) | NA | NA |
| rps15 | [92491:92757](+) | NA | NA |
| rps16 | [5259:5298](-) | [4649:4842](-) | NA |
| rps18 | [63231:64115](+) | NA | NA |
| rps19 | [78109:78582](-) | NA | NA |
| rps2 | [14364:15092](-) | NA | NA |
| rps3 | [76828:77499](-) | NA | NA |
| rps4 | [56480:57085](-) | NA | NA |
| rps7 | [107535:108005](+) | NA | NA |
| rps8 | [74128:74532](-) | NA | NA |
| ycf1 | [86648:92251](+) | NA | NA |
| ycf2 | [80004:86138](+) | NA | NA |
| ycf3 | [55373:55496](-) | [54386:54615](-) | [53460:53612](-) |
| ycf4 | [47319:47513](-) | NA | NA |
| trnH-GUG | [5:78](-) | NA | NA |
| trnK-UUU | [4223:4259](-) | [1674:1709](-) | NA |
| trnQ-UUG | [5568:5639](-) | NA | NA |
| trnS-GCU | [6906:6993](-) | NA | NA |
| trnT-CGU | [8074:8107](+) | [8762:8804](+) | NA |
| trnR-UCU | [8945:9016](+) | NA | NA |
| trnC-GCA | [25905:25975](+) | NA | NA |
| trnE-UUC | [28787:28859](+) | NA | NA |
| trnY-GUA | [28924:29007](+) | NA | NA |
| trnD-GUC | [29380:29453](+) | NA | NA |
| trnT-GGU | [29687:29758](+) | NA | NA |
| trnS-UGA | [33101:33189](-) | NA | NA |
| trnG-GCC | [34116:34186](+) | NA | NA |
| trnM-CAU | [34385:34456](-) | NA | NA |
| trnM-CAU copy2 | [39748:39820](+) | NA | NA |
| trnS-GGA | [56041:56127](+) | NA | NA |
| trnT-UGU | [57473:57545](-) | NA | NA |
